# Bringing together the World Health Organization's QualityRights initiative and the World Psychiatric Association's programme on implementing alternatives to coercion in mental healthcare: a common goal for action

**DOI:** 10.1192/bjo.2023.622

**Published:** 2024-01-05

**Authors:** Neeraj Gill, Natalie Drew, Maria Rodrigues, Hassan Muhsen, Guadalupe Morales Cano, Martha Savage, Soumitra Pathare, John Allan, Silvana Galderisi, Afzal Javed, Helen Herrman, Michelle Funk

**Affiliations:** School of Medicine and Dentistry, Griffith University, Australia; Mental Health Policy Unit, Health Research Institute, University of Canberra, Australia; and Mental Health and Specialist Services, Gold Coast Health, Australia; Department of Mental Health and Substance Use, World Health Organization, Geneva, Switzerland; Community Works, Docklands, Australia; and Kindred Collaborative, Brisbane, Australia; School of Medicine and Dentistry, Griffith University, Australia; and Mental Health and Specialist Services, Gold Coast Health, Australia; Fundación Mundo Bipolar, Madrid, Spain; School of Geography, Environment and Earth Science, Victoria University of Wellington, New Zealand; Centre for Mental Health Law and Policy, Indian Law Society, Pune, India; Mayne Academy of Psychiatry, School of Medicine, University of Queensland, Australia; Department of Mental and Physical Health and Preventive Medicine, University of Campania Luigi Vanvitelli, Italy; Pakistan Psychiatric Research Centre, Fountain House Institute, Lahore, Pakistan; Orygen, Parkville, Australia; and Centre for Youth Mental Health, The University of Melbourne, Australia

**Keywords:** Coercion, mental healthcare, human rights, involuntary treatment, mental health conditions

## Abstract

**Background:**

Stakeholders worldwide increasingly acknowledge the need to address coercive practices in mental healthcare. Options have been described and evaluated in several countries, as noted recently in major policy documents from the World Health Organization (WHO) and World Psychiatric Association (WPA). The WHO's QualityRights initiative promotes human rights and quality of care for persons with mental health conditions and psychosocial disabilities. A position statement from the WPA calls for implementation of alternatives to coercion in mental healthcare.

**Aims:**

We describe the engagement of both the WHO and WPA in this work. We discuss their mutual aim to support countries in improving human rights and quality of care, as well as the differences between these two organisations in their stated goals related to coercion in mental healthcare: the WHO's approach to eliminate coercion and the WPA's goal to implement alternatives to coercion.

**Method:**

We outline and critically analyse the common ground between the two organisations, which endorse a similar range of rights-based approaches to promoting non-coercive practices in service provision, including early intervention in prevention and care and other policy and practice changes.

**Results:**

Advocacy and action based on an agreed need to find practical solutions and advances in this area have the power to build consensus and unify key actors.

**Conclusions:**

We conclude that persons with lived experience, families, mental health professionals and policy makers are now coming together in several parts of the world to work toward the common goals of improving quality, promoting human rights and addressing coercion in mental health services.

The World Health Organization (WHO) and the World Psychiatric Association (WPA) recognise the important need to improve quality of care and ensure full respect for the human rights of people with mental health conditions and psychosocial disabilities, including the specific need to address coercion in mental healthcare.^1–4^ The WHO QualityRights initiative was created to shine a spotlight on this neglected area and to support countries in their efforts toward achieving these ends, including through the development of a range of resources and tools.^5–9^ The WPA has issued a position statement and set up a working group to support collaboration among actors and countries in implementing alternatives to coercion in mental healthcare.^1,4,10^ Reports from around the world indicate that coercive practices are still widespread in services everywhere.^11,12^ Coercive practices refer to the use of threat or compulsion to require a person to do something against their will. It includes the use of practices such as forced admission and forced treatment, as well as manual, physical or chemical restraint and seclusion.^1,9^

Many people subjected to coercive practices report experiencing them as a form of violence, trauma or re-traumatisation, leading to feelings of dehumanisation and disempowerment, and a worsening of their condition and increased distress.^13–16^ Coercive practices can undermine people's trust in mental health services, can cause harm to mental health and well-being, physical harm including death,^12^ and have negative consequences including moral injury for the well-being of the professionals using them.^17,18^ Many studies that have evaluated the effects of common coercive practices like community treatment orders, seclusion and restraint have either not shown them to be beneficial^19^ or have documented their negative effects.^20^ In light of this, a growing number of stakeholders worldwide acknowledges that there is a need to tackle and prevent coercive practices in mental healthcare as a key component of improving quality and increasing respect for human rights in health services.

In addressing coercion, it is crucial to understand and respond to the contextual factors and emotions that are leading to crises or challenging situations, and to create a more supportive environment to facilitate a person's recovery. Although there are different evidence-based approaches to implementing alternatives to coercion, as described throughout this paper, ultimately, at an individual level, it is crucial to communicate with the persons concerned to identify what non-coercive interventions can be helpful in crisis or challenging situations, and preferably before these develop. This approach is emphasised in the WHO QualityRights training materials and guidance on human rights-based community mental health services.^6,8,9^

The United Nations Convention on the Rights of Persons with Disabilities (CRPD) recognises this need to address coercion and requires major reforms and protection of human rights.^21^ It embeds the rights of persons with disabilities, including persons with mental health conditions and psychosocial disabilities, into international human rights law: requiring concerted actions by States Parties to prioritise and protect the rights to community inclusion, autonomy, citizenship and empowerment, and to access coercion-free services that respect their dignity and legal capacity. Since the adoption of the CRPD in 2006, several countries are seeking to align their laws, policies, services and practices with the Convention. However, there is a significant gap between what is mandated by the CRPD and the real situation on the ground. To date, few countries have established the necessary frameworks to meet the far-reaching requirements of the CRPD. It is noteworthy that there are different interpretations of the CRPD when it comes to involuntary psychiatric treatment.^22^ The CRPD Committee's authoritative interpretation has called for a total prohibition of all substitute decision-making regimes like involuntary psychiatric treatment and guardianship.^23^ This approach has received acceptance among many groups and stakeholders. However, other commentators have called for a less strict interpretation of the CRPD, arguing that Article 12^4^ allows for involuntary treatment as a last resort, for the shortest possible time, subject to safeguards and monitoring by a competent authority.^24,25^ Irrespective of this debate, there is general agreement that the CRPD provides a trajectory and horizon to recognise and tackle coercion in a way that respects the rights, will and preferences of people with psychosocial disabilities and mental health conditions.^26^ The CRPD also requires the States Parties to ensure an equality of rights enjoyment: that persons with mental health conditions and psychosocial disabilities are entitled to enjoy human rights on an equal basis with everyone else (with or without disabilities), and that the States Parties must provide the support that may be required to achieve such equality.

Over recent years, both the WHO and the WPA have sought to provide guidance and support to countries in addressing coercive interventions. Through its QualityRights initiative, the WHO promotes human rights and quality of care for people with mental health conditions and psychosocial disabilities, and has developed a range of resources and tools to support countries work toward eliminating coercion in mental health services.^7,27,28^ The WPA has published a position statement and a discussion paper on implementing alternatives to coercion in mental healthcare in 2020.^1,4,29^ These documents describe the debate about whether it is desirable to advocate for eliminating or for reducing coercion. The WPA has established a working group that continues to pursue its call to action by delivering educational sessions; publishing resources such as case studies; and engaging psychiatrists, people with lived experience of mental health conditions and family/informal carers in international consultations to inform future actions.^10^ Both the WHO QualityRights initiative and the WPA Implementing Alternatives to Coercion programme provide a direction and trajectory to protect human rights in mental healthcare, and improve respect for human rights and the quality of services for people with mental health conditions and psychosocial disabilities. It is worth noting, however, the inherent difference between the WHO QualityRights initiative approach to eliminate coercive practices and the WPA's call to implement alternatives. This paper articulates each of these two positions and seeks to reconcile them with a practical approach.

## WHO QualityRights initiative

The overall aim of the WHO QualityRights initiative is to promote a person-centred and human rights-based approach in mental health in line with the requirements of the CRPD and other international human rights standards. Efforts to eliminate forced admission and treatment including the use of seclusion and restraints, a goal in line with the United Nations authoritative interpretation of the CRPD, are important components of the QualityRights initiative as detailed in the discussion below.

Recognising the complexity of system issues contributing to poor quality of care and human rights violations, the QualityRights initiative works to influence change at several different levels: changing attitudes and mindsets to address stigma and discrimination, and promote understanding of human rights and reinforce practice change in the mental health context; building community-based services that are person-centred and rights-based, and avoid the use of coercive practices; engaging civil society, in particular people with lived experience, in decision-making and actions related to mental health; and developing new policy and law frameworks that reinforce an integrated rights-based approach that includes provisions and actions to eliminate coercive practices.

Through the QualityRights initiative, the WHO has developed a range of training and guidance materials and tools to support national efforts in this direction. One set of tools includes QualityRights face-to-face training modules on key issues and approaches required for the elimination of coercive practices from the mental health sector.^6^ Although these have been developed to provide intensive training to key stakeholder groups within the mental health system, the WHO has also created an e-training programme on mental health, recovery and community inclusion.^8^ The latter covers the same ground as the face-to-face materials, but is designed to enable the training of large segments of the population on rights-based, person-centred approaches to mental health, and thereby influence societal mindsets at large.

A second set of tools directly addresses the need for countries to establish rights-based, coercion-free, community-based mental health services. Indeed, the WHO guidance and technical packages on human rights-based community mental health services released in 2021 describe 28 services from low-, middle- and high-income countries that provide quality care and support, as well as uphold key human rights principles of legal capacity, non-coercion, participation and community inclusion by using a combination of strategies outlined in the WHO QualityRights face-to-face training materials and the e-training as described below. Good practices are showcased for the main categories of community-based services: crisis services, hospital-based services, community centres, peer support services, community outreach services, supported living and comprehensive mental health service networks. The overall guidance and technical packages also provide information and recommendations to countries on how to develop and scale up coercion-free and person-centred services: services that align with the rights of the CRPD and that achieve good health and social outcomes, often at comparable or lower costs than the existing mainstream provision.^9^

Some countries need to develop new services from scratch. Other countries might seek to transform those existing into good-practice, coercion-free services. In either case, the WHO QualityRights assessment toolkit and accompanying guidance on transforming services and promoting human rights enable countries to assess and improve their services in line with human rights standards. These resources address areas related to freedom from coercion, recognising legal capacity, informed consent to treatment, supported decision-making, advance directives and community inclusion, among others.^5,30^ Coercion can result from, and/or is reinforced through, the culture and power imbalances within mental health services. The WHO transformation guidance helps to explore how these imbalances can be addressed, and how core values such as equality, respect and dignity can be embedded in the service provided. This WHO tool also provides guidance on using a participatory approach to work through the specific priorities identified during the service assessment, and develop an action plan to address these.

The QualityRights initiative recognises that undertaking rights-based reform to eliminate coercion in services is a challenging undertaking, particularly when policy and law frameworks are legitimising and reinforcing these very practices. Furthermore, stigma and risk aversion in services and society can usurp progressive frameworks. New WHO policy and law guidance is underway to provide a new rights-based framework that has at its core the respect for legal capacity, community inclusion and participation, and access to coercion-free mental health systems and services.

Persons with mental health conditions and psychosocial disabilities are involved in all aspects of the WHO QualityRights initiative, including the development of the QualityRights normative tools, training and capacity-building actions, and the design and implementation of the QualityRights initiative at the country level. Their participation and contribution toward the QualityRights initiative has brought meaning and acceptance, and had a demonstrable impact. Furthermore, the WHO QualityRights initiative recognises that change requires the participation of all stakeholder groups, including those in the justice system (for example, the police and first responders to crisis situations), as well as action in all contexts, including those where high levels of coercion are usually mandated (for example, forensic facilities). Several studies have shown that even in these latter settings, eliminating coercion has positive effects.^31–33^

## The WPA Position Statement on Implementing Alternatives to Coercion in Mental Healthcare

The WPA in 2020 adopted the ‘Position Statement on Implementing Alternatives to Coercion In Mental Healthcare’, with an emphasis on the protection and promotion of human rights.^1,4^ This was the culmination of the work of a taskforce set up by the WPA, in a joint project with the Royal Australian and New Zealand College of Psychiatrists. The taskforce aimed to provide guidance to 140 member societies of the WPA in supporting collaborative approaches to implementing alternatives to coercion.^1,29^

The taskforce began by commissioning a literature review and discussion paper on ‘Minimising Coercion in Mental Health Care’. Through discussion with human rights advocates, psychiatrists, people with lived experience of mental health conditions and family/informal carers, however, it soon became clear that the stated objective of minimising coercion was divisive because some advocates insisted on complete elimination rather than minimisation of coercive interventions, whereas others maintained the need for occasional use of coercive interventions as the last resort for a short period of time to promote the right to health. The focus of the work was redirected toward a common overarching goal that would address the primary need emerging from the review: implementation of alternatives to coercion. The discussion paper categorises relevant research from different socioeconomic and cultural contexts on alternatives to coercion. The categories include initiatives for change at the service level (hospital and community settings), regional initiatives, and national and legal policy change. Several strategies and examples of implementing alternatives to coercion in clinical and service settings are highlighted, in particular ‘Safewards’,^34^ ‘Six Core Strategies’,^35^ the ‘Open Door Policy’^36,37^ and WHO QualityRights resources described above, as promising ways of improving the human rights compliance of a mental health service. The discussion paper provides an evidence-based resource to inform ongoing debate within and beyond the psychiatric profession, with emphasis on implementing alternatives to coercion and identifying opportunities to trial promising initiatives in different settings.

The WPA member societies were consulted on the validity, importance and feasibility of implementing the alternatives to coercion presented in the paper, considering the policies, practices and experiences in their own countries and regions. Responses were received from 16 national psychiatric societies and associations in eight distinct geopolitical regions (Eastern Europe, Western Europe, North America, South America, Middle East, South Asia, East Asia and Australia Pacific). A parallel consultation was held with patients and family/informal carers. The report of that consultation was considered alongside survey responses from member societies.

A thematic analysis was conducted to identify implications for developing the position statement. These included: (a) psychiatrists, persons with lived experience of mental health conditions and family/informal carers across all responding geopolitical regions recognise the importance of implementing alternatives to coercion to protect human rights and empower people with mental health conditions and psychosocial disabilities; (b) strong relevance of this work in low- and middle-income countries, which face complex systemic barriers; (c) alignment with transitions to recovery-oriented and trauma-informed models of treatment and care; (d) the alternatives reviewed by the paper are being implemented in a variety of different social, cultural and economic contexts despite significant resource barriers and urgent need for improvement in all of these settings; and (e) there is a clear need for cultural change, early intervention and involvement by people with lived experience in research, policy and practice. Overall, the consultation found broad support for the position expressed in the discussion paper and strong potential for the positive framing of ‘implementing alternatives’ as an effective way of unifying psychiatrists, persons with lived experience and family/informal carers toward movement in a common direction, even where there is scepticism about the ultimate goal of elimination.

Guided by the evidence base presented in the discussion paper, paired with consultation findings, the position statement begins by defining coercion and outlines different types of traumas associated with the experience of coercion in mental healthcare. The accompanying discussion paper elaborates on different forms of coercion and the clinical, moral and legal grounds and motivations for finding and implementing alternatives to coercion.^29^ Both documents acknowledge the range of views on the feasibility of eliminating coercion, discuss socioeconomic and cultural barriers, and put forward recommendations for implementing safe and high-quality alternatives. The documents represent a wide scope of perspectives among clinicians, people with lived experience and their families/informal carers and representative bodies on implementing alternatives to coercion, recognising the divergence of post-CRPD interpretations on these critical topics.^22,24^

The WPA position statement recommends augmenting research into alternatives to coercion. It urges priority for developing and testing alternatives to coercion, as well as adapting existing resources to implement alternatives. In doing so, it addresses the significant need to diversify the evidence base and generate a better understanding of barriers to change and the consequences of change. The statement recommends engaging with people with lived experience and their families and informal carers, to bring lived experience and insight into research plans and proposals, and encourages the sharing of experience across settings and countries. It further calls upon researchers to address coercion in community settings, especially in countries with a dearth of mental healthcare and support.

The position statement calls for practitioners, including psychiatrists and especially those in leadership roles, to partner with patients and their families to advocate for and enact changes in clinical, political and cultural settings.^38^ In clinical settings, it encourages practitioners to make use of evidence-based resources, ensure adequate training is provided to staff in delivering non-coercive care and understand how best to influence attitudes and practices in interacting with people with lived experience and their families. It also calls on practitioners who are in a position to do so to take an active role in persuading policy makers to prioritise the implementation of alternatives to coercion, provide adequate public resources, establish databases to record instances of coercion and support early intervention in episodes of mental ill health. More broadly, it encourages practitioners to work with government agencies and professional organisations to shift professional norms on the use of coercion and raise awareness about alternatives. It also identifies stigma toward people with mental health conditions and psychosocial disabilities as a fundamental contributor on service providers to overuse coercion.^39^ It calls on psychiatrists, other practitioners, people with lived experience and their families and informal carers to work with media and politicians to counter this stigma, and encourage a culture of participation in mental healthcare for all stakeholders.

In 2021, the WPA established a working group in collaboration with a number of member societies, ‘Supporting and Implementing Alternatives to Coercion in Mental Health Care’, to engage psychiatrists and other partners in taking active steps toward actualising the recommendations put forward in the position statement.^10^ In recognition of the importance of involving people with lived experience of mental health conditions and family/informal carers, the working group collaborates closely with the WPA working group, ‘Developing Partnerships with Service Users and Family Carers’. Four people are members of both working groups, including one patient and one family member advocate, both of whom are connected to extensive international peer networks. This setup has facilitated meaningful participation of persons with lived experience, transparency and respectful debate, as the two groups continually find ways of working together. The WPA has established a dedicated section of the WPA website, where resources, initiatives and links to useful materials can be found;^40^ has documented case studies, such as the QualityRights Initiative in Gujarat, India;^41^ has organised a course on alternatives to coercion; and has initiated two international consultative surveys, one for WPA member societies and one for persons with lived experience and carers, to inform future directions of this work.^10,42^

## Discussion

Both the WHO and WPA recognise the critical need to address coercion in mental health service settings. From the WHO perspective, it is essential that the goal be to eliminate all coercive practices, and that countries set this as their vision and target. The WPA calls for alternatives to coercive practices that will increase observance of human rights and improve quality of care in mental health services.

Arguments in favour of advocating for, and working toward, reducing rather than eliminating coercive practices are based on several lines of reasoning. It is argued that ‘exceptional’ measures, such as guardianship and involuntary admission, treatment, seclusion and restraint, are sometimes necessary to prevent danger to one's self or others, and to ensure that people receive the care and support they need.^43^ It is also argued that, although supported decision-making provisions need to be strengthened, involuntary treatment is sometimes necessary as a last resort, for the shortest possible time, with strong safeguards in place, to balance the competing sets of rights (e.g. right to autonomy and right to health).^25,44^ The difficulty of eliminating coercive practices is highlighted by the fact that some services showcased in the WHO's guidance and technical packages on rights-based community mental health services had referred a minority of patients to other services where coercive practices still operate, when confronted with situations they deemed too challenging. However, it is important to acknowledge that the good practice services showcased in the WHO guidance are operating within systems that are not aligned with current human rights standards mandated by the CRPD. The WHO argues that if services were able to function within a framework in which polices, laws, attitudes and mindsets were aligned with these standards, persons posing safety issues or experiencing challenging acute crises could be accommodated and supported non-coercively.

The WHO's approach to eliminate coercion aims to avoid the situation in which ‘exceptions’ become the rule, and to ensure that coercive practices are seen as something negative and to be avoided. The WHO recognises that, because of limitations in current mental health systems and difficult contexts, involuntary practices may still occur even in situations where staff have made great efforts to implement alternatives to coercion. However, with the ‘elimination’ approach, it is essential that the use of any coercive practices is seen as an opportunity for review and learning, and that measures are put in place to avoid their use in the future. In this way, this approach enables services to always strive to do better, as well as to address the system-level issues that act as important barriers to the elimination goal.^45^

The WPA position calls for practical system and service changes that support alternatives to coercion. The WPA explicitly avoids advocacy for eliminating coercion, based on the view that although individual autonomy, will and preferences must be respected, there are specific occasions when coercion, including involuntary psychiatric admission, is needed to promote both safety and the right to health, where less restrictive interventions cannot achieve that.^46–48^ However, the word ‘minimising’ was not acceptable to many stakeholders, and hence the WPA emphasised the importance of implementing alternatives to coercion in mental healthcare. This is designed to support psychiatrists and other practitioners to work in good conscience toward pragmatic changes within the systems in which they find themselves, whatever their views on the ideological questions and whatever the level and types of resources and training extant in the service setting.

Even the United Nations human rights system has been divided in opinion on whether coercive psychiatric interventions can ever be justified under the contemporary human rights standards.^49^ Apart from this disagreement, sometimes known as the ‘Geneva impasse’, the WHO and WPA share much common ground. Both recognise that to address coercive practices in mental healthcare, a number of significant barriers need to be overcome. These barriers include lack of adequate financial and organisational investment in services, including perverse incentives for perpetuating coercion; outdated undergraduate and graduate curricula for healthcare and other practitioners; lack of training in alternatives to coercion; outdated mental health-related policy and laws that perpetuate coercion; and the lack of research on alternatives to coercive practices.

Key recommendations are mentioned in the WPA and WHO documents cited above, and elaborated on comprehensively in the 2021 ‘WHO Guidance and Technical Packages on Community Mental Health Services: Promoting Person-Centred and Rights-Based Approaches’. They are summarised and supplemented below.^9^

### Addressing financial and organisational factors

Allocating sufficient financial resources is a necessary precondition for developing high-quality mental health services that adequately respond to and meet people's needs. But what is essential is that investment is directed toward the right kind of services, those that are based in the community, rights-oriented and person-centred at their core, and actively implement alternatives to coercion.^9^ The centre of gravity of mental health services must shift from hospitals to community-based care, with well-resourced and systematised voluntary alternatives to promote a recovery and rights-based orientation.^50^ In recent years, an increasing number of such services have been emerging in different parts of the world, documented in various articles and reports, including the WHO's 2021 guidance on rights-based community mental health services highlighted above.^9,49,51^

Together with investing in services, efforts to reduce coercion need to address organisational and environmental factors within services.^52^ Pre-empting and managing tense, difficult or conflict situations, for instance, requires sufficient numbers of properly trained staff, adequate staff supervision, service-level policy or protocols that focus on promoting alternatives to coercion and putting in place effective mechanisms to monitor human rights conditions in mental health services.^53,54^ Service investment and training also need to be oriented toward early intervention and support, and toward collaborative care to underpin recovery in the community.^55^ These service orientations have an important role in averting the worsening of emotional distress and crises, and in turn, the circumstances in which coercive practices are more likely to be used.

### Training and education

Redesigning undergraduate and graduate course curricula in nursing, medicine, psychology, social work and occupational therapy, among other areas, is another critical strategy to promote alternatives to coercion in mental health services. Current educational and training curricula, which include coercive practices and often present them as a necessary part of mental health practice, need to be replaced and refocused to incorporate education and training on human rights, disability and person-centred recovery approaches in mental healthcare.^9,53^ The human rights discourse must be integrated into the training, practice and language of psychiatry.^44^ The WPA ‘Code of Ethics for Psychiatry’ recognises that optimal psychiatric care is rendered through collaboration among patients, carers and clinicians and other team members.^47,56^ Inclusion of persons with lived experience and their family and informal caregivers in developing and delivering training is recommended.

Capacity-building to shift attitudes and practices is important not only for undergraduate and graduate students, but also professionals and their leaders already active in mental health services.^9^ Furthermore, both students and professionals should receive training specifically on alternatives to coercion. Both the WHO QualityRights face-to-face training materials and the e-training described above build knowledge and skills on how to end coercive practices. Different strategies are introduced, such as understanding and addressing power dynamics within mental health services, developing individualised plans to explore and respond to sensitivities and signs of distress before any potential crisis emerges, putting in place advance directives and supported decision-making mechanisms, creating a ‘saying yes’ and ‘can-do’ culture to avoid frustrations from intensifying, supportive environments and the use of comfort rooms, de-escalation of tense and conflictual situations, and trained response teams that intervene during crises without force.^27,30,53,57,58^

### Mental health-related policy and law

Outdated policies and laws related to mental health act as barriers to addressing coercion by serving to maintain, regulate and perpetuate coercive services, systems, structures and practices. A major step on the path toward promoting alternatives to coercive practices is therefore ensuring that mental health policies and plans explicitly promote a shift toward comprehensive, person-centred, recovery-oriented and holistic services that respect people's will and preferences in treatment.^1,9^

The primary function of mental health laws in most countries is to authorise and regulate coercive practices including involuntary admission and treatment, as well as seclusion and restraint. In addition, substitute decision-making legislation (including guardianship and capacity-related laws) aim to regulate restrictions in people's right to exercise their legal capacity. A significant effort is needed by countries to bring national legal frameworks in line with CRPD standards. This means developing laws that recognise people's right to the full and effective exercise of their legal capacity; to informed consent to treatment and to access supported decision-making, advance directives and other measures that help to promote legal capacity and alternatives to coercive practices. Legislation concerning medical liability or medical malpractice should also be reformed so that practitioners are not put in the position of resorting to the use of coercive practices as a means to avoid risk of harm, and can instead keep focus on measures that ensure respect for people's rights.^9,59^ Although legislation provides impetus for change, regulatory frameworks that monitor and implement alternatives to coercion – such as mental health review bodies, national human rights institutions, national preventive mechanisms and bodies tasked to monitor the implementation of the CRPD – are still practically necessary to bring systemic changes. In addition to laws addressing coercion head on, it is also important for legislation and policy to address the many social and structural determinants and discriminatory factors that negatively affect mental health, precipitate crisis situations and act as barriers to people's recovery. Provisions need to address economic, social and cultural rights, including access to quality health and mental health services and supports, social services, social protection, employment, education, housing and other relevant areas.^60,61^

### Research on alternatives to coercion

The major gap that exists in research on non-coercive interventions, practices, services and supports also acts as a significant barrier to change. Increased investment and funding are needed for both quantitative and qualitative research and evaluations. Examples are research on implementing CRPD-aligned services and on measures and practices within services to avoid the use of coercive practices, including de-escalation, the deployment of response teams, different models of supported decision-making interventions, advance directives and peer support, as described in the WHO guidance and technical packages on human rights-based community mental health services.^1,9^ It is critical that experts by lived experience contribute to and lead in the design and implementation of research, as well as in all other reform endeavours.^9^ To judge impact and effectiveness, consistent reporting mechanisms for coercive practices and alternatives to coercion need to be implemented across all countries. Current reporting of coercion is highly inadequate.^11,62,63^ Mechanisms need to be put in place to assess the median duration and average number of mechanical and physical restraints and seclusion, involuntary hospital admission and treatment, including forced medication.

In conclusion, regardless of whether the stated aim is complete elimination of coercion or finding alternatives to these practices, the major challenges and barriers outlined above need to be overcome. Rather than being stalled by debate about the stated aims, both the WHO and WPA are calling for all countries to take concrete action at policy, service and clinical practice levels to promote non-coercive practices, care and support that respect people's rights, dignity and choice. Mental health professionals, persons with lived experience, families, civil society actors and other actors including, and in particular, those in leadership positions should consider how they can actively contribute to change. Change is needed to uphold human rights and improve the quality of mental healthcare, making full use of the WHO QualityRights tools and training materials and the WPA resources highlighted in this paper.

## Data Availability

Data availability is not applicable to this article as no new data were created or analysed in this study.
